# Epi-Impute: Single-Cell RNA-seq Imputation via Integration with Single-Cell ATAC-seq

**DOI:** 10.3390/ijms24076229

**Published:** 2023-03-25

**Authors:** Mikhail Raevskiy, Vladislav Yanvarev, Sascha Jung, Antonio Del Sol, Yulia A. Medvedeva

**Affiliations:** 1 Department of Biological and Medical Physics, Moscow Institute of Physics and Technology, 141701 Moscow, Russia; 2 Skolkovo Institute of Science and Technology, 121205 Moscow, Russia; 3 Computational Biology Laboratory, Center for Cooperative Research in Biosciences, 48160 Derio, Bizkaia, Spain; 4 Centre for Systems Biomedicine, The University of Luxembourg, 4365 Luxembourg, Luxembourg; 5 Institute of Bioengineering, Research Center of Biotechnology, Russian Academy of Sciences, 119071 Moscow, Russia; 6 The National Medical Research Center for Endocrinology, 117036 Moscow, Russia

**Keywords:** single cell RNA-seq, imputation, single cell ATAC-seq

## Abstract

Single-cell RNA-seq data contains a lot of dropouts hampering downstream analyses due to the low number and inefficient capture of mRNAs in individual cells. Here, we present Epi-Impute, a computational method for dropout imputation by reconciling expression and epigenomic data. Epi-Impute leverages single-cell ATAC-seq data as an additional source of information about gene activity to reduce the number of dropouts. We demonstrate that Epi-Impute outperforms existing methods, especially for very sparse single-cell RNA-seq data sets, significantly reducing imputation error. At the same time, Epi-Impute accurately captures the primary distribution of gene expression across cells while preserving the gene-gene and cell-cell relationship in the data. Moreover, Epi-Impute allows for the discovery of functionally relevant cell clusters as a result of the increased resolution of scRNA-seq data due to imputation.

## 1. Introduction

Single-cell RNA sequencing (scRNA-seq) has revolutionized cell biology, enabling studies of heterogeneity and transcription dynamics of complex tissues at the highest resolution [1]. However, the method has significant limitations due to the high sparsity of generated data. This effect is usually attributed to a “dropout” problem [2], resulting in capturing only a small fraction of each cell transcriptome. The excessive zero counts cause the scRNA-seq data to be zero-inflated [3]. Moreover, dropouts usually combine two distinct types of zero values: those caused by technical limitations and those caused by a biologically relevant absence of expression [4]. Technical limitations can be attributed to a platform and sequencing depth [5,6], while biological limitations arise from low amounts of mRNA molecules in a single cell and stochastic gene expression [7]. All these issues lead to a large share of genes with high expression in one cell and zero expression in another cell of the same type. This problem gives rise to biases in computational downstream analysis, including clustering, classification, differential expression, and gene-regulatory network reconstruction [4,8,9].

Several imputation approaches have been recently developed to address this critical issue. MAGIC [10] imputes dropouts by data diffusion based on a Markov transition matrix that defines a kernel distance measure among cells. scImpute [11] estimates dropout probability using a two-component mixture model and applies a LASSO model to impute dropouts. SCRABBLE [12] performs matrix regularization for imputing scRNA-seq data using bulk RNA-seq as a constraint. Lun et al. [13] proposed a pool-and-deconvolute approach to deal with dropouts for imputation and normalization. Similarly, SAVER [14] also relies on linear regression to impute missing data, yet it implements a Bayesian model to compute the probability of dropout events. DrImpute [15] performs consensus clustering of cells followed by imputation of average expression values in similar cells. DCA performs denoising using a negative binomial noise model with or without zero-inflation and nonlinear gene-gene dependencies [16].

Even though these methods improve downstream analysis, they rely solely on RNA-seq data making strong assumptions about statistical distributions for gene expression and dropout probabilities resulting in high false-positive rates, i.e., an expression value being imputed for a gene that is not expressed [17]. A major challenge of all existing imputation approaches is the circularity arising from only internal information used for imputation [4]. This circularity can artificially amplify the signal contained in the data, leading to inflated correlations between genes or cells and, as a result, to a lot of false positives in downstream analyses [17].

Single cell ATAC-seq (scATAC-seq) is a method capable of revealing active regulatory regions—promoters and enhancers [18,19]—and shows a strong correlation with gene expression. As well as any single-cell-based method, scATAC-seq suffers from relatively low read counts per cell and biases shared with other sequencing-based methods [20]. Yet, being based on sequencing of a more stable DNA molecule and therefore not affected by RNA degradation, scATAC-seq can compensate for missing gene expression values in scRNA-seq data.

Here, we present Epi-Impute, a computational tool for imputing scRNA-seq data from DNA accessibility data (scATAC-seq) from consistent cell-type populations. Epi-Impute is the first imputation method that incorporates epigenetic information into the imputation procedure. It outperforms existing imputation methods, even those using external data, while preserving the original gene-gene and cell-cell relationship in the expression data. Additionally, Epi-Impute allows the discovery of new functionally meaningful cell clusters in the downstream analysis thanks to the increased resolution of scRNA-seq data caused by the imputation.

## 2. Results

### Epi-Impute Pipeline

**Figure 1:**
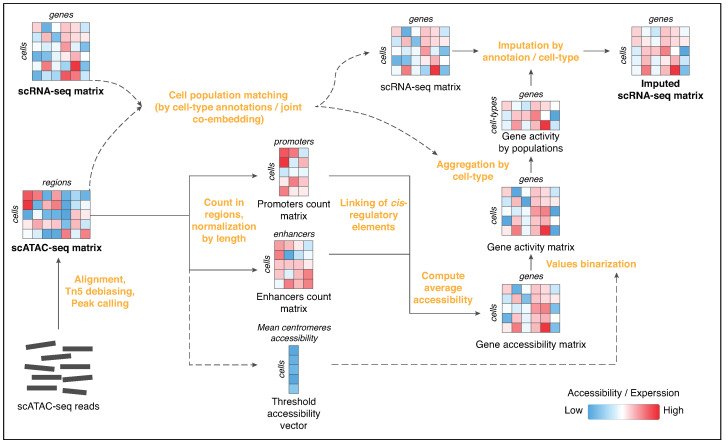
An overview of the Epi-Impute pipeline. Data processing steps are highlighted in orange. Average accessibility in centromeres regions from ENCODE Blacklist [21] was used to create a Threshold accessibility vector to assess signal-to-noise ratio of chromatin accessibility.

To compensate for dropouts in scRNA-seq, Epi-Impute estimates a probability of a gene being expressed based on the average accessibility of gene regulatory elements (e.g., promoters and enhancers) across a cell type (Figure 1). Of note, we assume that cells in both scRNA-seq and scATAC-seq data are already annotated and matched using FACS surface markers or co-embedding prior to the Epi-Impute procedure.

After scATAC-seq reads alignment and *Tn5* debiasing, Epi-Impute creates a threshold accessibility vector for scATAC-seq data using heterochromatic centromeric and telomeric regions from the ENCODE blacklist [21] (https://github.com/raevskymichail/genome_browser_storage/blob/master/ucsc_hg38_centromeres.tsv, accessed on 15 March 2023), as well as count matrices for *cis*-regulatory elements (promoters and enhancers separately) of the genes. More specifically, Epi-Impute counts ATAC-seq reads aligned within the *–500/+200  bp* window around a TSS or an enhancer and applies a smooth quantile normalization [22] and GC-content normalization using the CQN (Conditional Quantile Normalization) R-library [23]. A TSS or enhancer in a cell is considered to be open if its coverage is above the corresponding value in a threshold accessibility vector. Then, a gene accessibility matrix is obtained from the previous step if any of its regulatory elements (promoter or enhancer) is detected to be open in a given cell. Next, a gene accessibility matrix for each cell type is binarized using provided or obtained from scRNA-seq/scATAC-seq co-embedding cell annotations and a threshold accessibility vector with a sigmoid function. Thus, a gene activity matrix is obtained, which reflects a probability of a particular gene to be expressed based on the accessibility of its *cis*-regulatory elements. The gene activity matrix is then aggregated per cell type (cluster) by calculating median values for each cell type (cluster), resulting in a gene activity matrix by cell populations, which is added to each gene expression count for each cell of a specific cell-type (cluster) in the scRNA-seq matrix (Figure 1).

**Figure 2:**
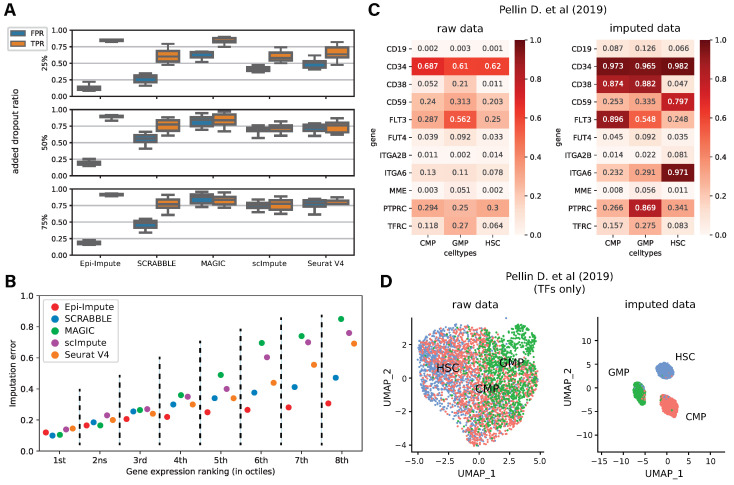
Epi-Impute performance: (**A**) True positive (TPR) and false positive rates (FPR) estimated based on the recovery of cell-type surface markers on an aggregated dataset, collected from Tabula Muris, PBMC 10x datasets and some GEO datasets (Supplementary Table S1, 9 datasets aggregated). Top to bottom: TPR and FPR for each of the methods tested on the series of data sets with a given ratio of simulated drop-outs. Each boxplot denotes a distribution of the performance metric obtained across all cell types presented in the series of data sets. (**B**) Imputation error (1−F1 score) at various expression levels on an aggregated dataset. (**C**) A share of cells in an annotated cluster expressing known cell-type-specific surface markers for this cluster: raw (left panel) and imputed data (right panel) from Pellin D. et al. [25]. (**D**) UMAP clustering based on transcription factors expression for raw (left panel) and imputed data (right panel) from Pellin D. et al. [25].

Epi-Impute utilizes the idea that chromatin is lightly packed in the regions within *cis*-regulatory elements of actively expressed genes; in other words, Epi-Impute uses chromatin accessibility to estimate a probability of a gene being expressed. Surface markers are generally used to identify a cell population during FACS sorting. To validate our approach, we compared *True positive (TPR)* and *False positive rates (FPR)* based on positive and negative FACS surface markers (Supplementary Table S3) across several imputation methods while increasing the number of simulated dropouts introduced to real transcriptomic data (“Marker test”). We assume that the presence of a surface marker in a cell should be reflected in its gene expression in the same cell. In this way, a *True positive* class is defined as all non-zero expression values observed for positive surface markers, and a *True negative* class is defined as all elements with zero expression for negative surface markers in the scRNA-seq matrix (Figure 2A, Supplementary Figure S3).

An alternative approach to defining *True positive* and *True negative* classes is to consider the most expressed genes in the bulk RNA-seq as a positive class and zero-expressed genes as a negative class for the matching cell population (“Bulk RNA-seq test”). This approach relies on the observation that with the increase of read coverage, the bulk RNA-seq data achieves saturation, meaning that almost all mRNA are captured [24]. Since some single-cell imputation methods were validated on bulk RNA-seq data, we also performed validation for Epi-Impute and the same imputation methods using a data set with various shares of simulated dropouts (Supplementary Figure S2). It is of note that for benchmarking, we used the same matched bulk RNA-seq data as for imputation with SCRABBLE [12].

Both “Marker” and “Bulk RNA-seq” tests show that Epi-Impute accurately recovers genes expression in terms of TPR and FPR even when dropouts levels were above 50%, suggesting that Epi-Impute introduced less bias while recovering genes expression values in a very sparse dataset.

Next, we tested to what extent Epi-Impute and other methods were prone to imputation errors in the most difficult case of low-expressed genes. As expected, all methods demonstrated an increased imputation error in the case of low-expressed genes (Figure 2B). Yet, as compared to other methods Epi-Impute shows the smallest imputation error when recovering low-expressed genes.

Subsequently, we focused on the robustness of the recovery of cell-type-specific surface markers by Epi-Impute and other methods. Since marker-specific antibodies were used in the original FACS sorting, we expected these markers to be expressed across all cells of a specific cell type. Epi-Impute was able to accurately recover the expression of surface markers reflecting FACS antigen-based definition of a given cell type (Figure 2C). Notably, in the case of positive surface markers, Epi-Impute recovers dropouts in the vast majority of cell populations. For HSC positive markers (*CD34, CD59, CD49f/ITGA6*), expression was recovered for 79.7% of cells. Similarly, Epi-Impute correctly imputed expression of cell-type selective markers, such as *CD135/FLT3*—a positive marker for CMP and a negative marker for GMP—and *CD45RA/PTPRC*—a positive marker for GMP and a negative marker for CMP—in more than 86.9% of the corresponding cell populations. Subsequent GO analysis confirmed the functionality of the imputed genes (Supplementary Figure S4). On the contrary, imputation with other methods resulted mostly in non-cell-type-specific marker expression (Supplementary Figure S5).

Next, we investigated how Epi-Impute can improve downstream analysis. Clustering cell populations based on the expression of regulatory genes benefit from the simultaneous detection of key regulators for cell populations. However, the clustering of highly homogeneous cell populations using mostly low-expressed regulatory genes is incredibly tricky [26,27]. Epi-Impute allows clustering of closely related populations, such as hematopoietic progenitors, using functionally important but low-expressed genes, such as transcription factors (Figure 2D, Supplementary Figure S6) while preserving gene-cell expression patterns primarily observed in raw data. At the same time, Epi-Impute does not introduce false batch patterns that can be treated by the clustering algorithm as the main components for dimension reduction (Supplementary Figure S7).

We also assessed to what extent the methods preserve the original gene-gene relationship. We used levels of fluorescence from RNA FISH and Drop-seq data for seven pairs of the housekeeping genes [28]. We performed a correlation analysis between the imputed expression profiles obtained by different methods for housekeeping genes in Drop-seq data and compared the results to the Pearson correlation (*r*) obtained for RNA FISH data as a “ground truth” (Table 1). All but two methods (MAGIC and scImpute) demonstrate comparable results. For example, the “ground truth” correlation between housekeeping genes *BABAM1* and *LMNA* observed within RNA FISH data is r=0.67 while for Epi-Impute r=0.68. A similar tendency for the other six gene pairs is provided in the Supplementary File S1.

**Figure 3:**
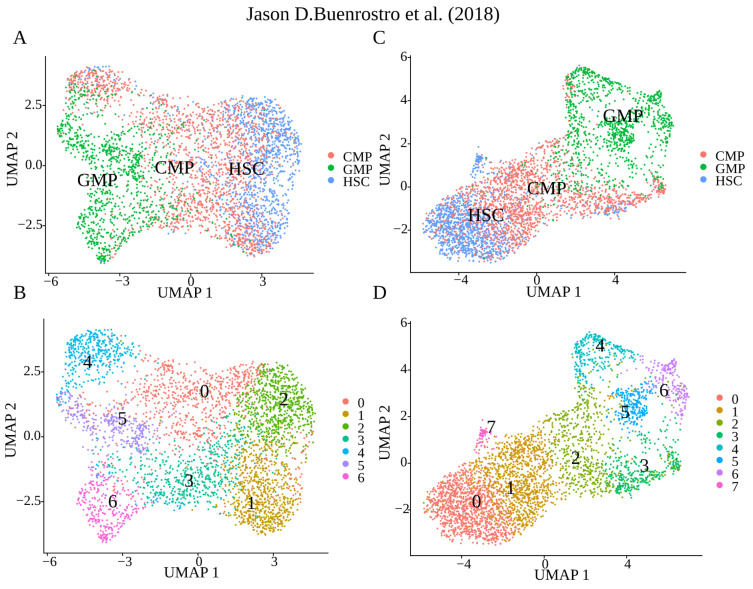
Epi-impute effect on clustering [18]: (**A**) Epi-impute imputed dataset, cell types determined based on surface markers. (**B**) Epi-impute imputed dataset, de novo clusters obtained by Seurat. (**C**) Raw dataset, cell types determined based on surface markers. (**D**) Raw dataset, Image shows the division de novo clusters obtained by Seurat.

Finally, we performed clustering of raw data and the data imputed by different methods to assess how imputation affects the resulting cluster structure. For the majority of methods, the UMAP cluster structure has not been changed dramatically (Supplementary Figure S8). Due to the high homogeneity of the initial data, imputation by all methods changed the clustering only slightly (Supplementary Table S4). Yet, in the case of Epi-Impute, a UMAP with the default parameters explicitly allocated to cluster 4 (Figure 3B). Differential analysis of this cluster against the two nearest ones (clusters 1 and 5) and against all other clusters detected *ENO1*, *MIF*, *DUT*, *SNRPB*, *KPNB1* and *NUCKS1*, *DUT*, *TUBB*, *TUBA1B*, *TYMS*, as upregulated in cluster 4, respectively. PAL analysis demonstrated increased activity in the cellular pathways responsible for the cell cycle (Supplementary Figure S9), for example, “Reactome Unwinding of DNA Main Pathway” and increased activity in the *GINS1/2/4* and *CDC4* genes (Supplementary Figure S10). These genes are essential to the DNA replication process in eukaryotic cells. Summing up, imputation leads to a more detailed cluster structure allowing cells in the active phase of the cell cycle to form a separate cluster.

## 3. Discussion

In this paper, we propose an imputation method for scRNA-seq data based on assessing gene activity from the chromatin condition of the gene regulatory region. We emphasize that measuring gene expression and chromatin accessibility in the same cell is still technically challenging. Therefore, our method integrates gene expression and chromatin accessibility measured for the same cell types based on a prior cell type annotation in both scRNA-seq and scATAC-seq or for similar cell populations linked to each other by any integration method using graph embedding. In this way, we avoid technical complications and reduce unwanted bias from each information source during imputation. Subsequently, our method does not affect cell type identification but instead improves recovery of gene expression in the cells of matching cell type.

Since Epi-Impute is the first tool using various data for imputation (scRNAseq and scATACseq), the comparison with other imputation methods is not entirely fair. It is only natural that more data of different natures contributes to improved imputation accuracy. Yet, the other imputation methods aim to solve the same problem, making benchmarking reasonable. We stress that Epi-Impute, an approach based on two data types, outperforms other existing imputation methods based solely on one data type capturing the primary distribution of gene expression across cells while preserving the gene-gene and cell-cell relationship in the data.

We emphasize that Epi-impute relies on enhancer boundaries detection based on prior knowledge since direct detection of enhancers with the use of the scATAC-seq data might be tricky due to data sparsity. On the other hand, scATAC-seq data allow us to estimate if a given enhancer is open and, therefore, available for the TF to bind. We use enhancer annotation obtained based on the bulk RNA-seq CAGE data for humans [29]. This approach does not only allow us to find enhancers everywhere in the genome but also to link an enhancer to a regulated promoter based on the co-expression analysis. In the future, single-cell Hi-C data might prove useful in recovering enhancer-gene links, but these data are still much less common now than the scATAC-seq data and, therefore, unpractical. There is one possible limitation to the use of scATAC-seq data for checking out if a particular enhancer is open or not. It is well accepted now that enhancers may open prior to promoter activation [30]. Yet, we believe that it is a more common scenario for differentiation, while for mature cells, there will be no major time lapse. Summing up, using enhancer expression as a proxy of promoter expression might be a simplification, but we find it reasonable and very practical.

In the benchmark study, Epi-Impute demonstrates both high accuracy and stability of imputation across data sets even in case of high sparsity of scRNA-seq matrix, without generating a large number of FP, staying both sensitive and selective, in contrast to slightly more sensitive methods such as MAGIC and SCRABBLE. As expected, SCRABBLE demonstrates FPR=0 in tests where classes were defined using bulk RNA-seq of matched cell populations (Supplementary Figure S2) since the algorithm utilizes the bulk RNA-seq data for imputation. Being tested on independently defined classes based on surface markers, SCRABBLE does not reproduce such dramatically low FPR (Supplementary Figure S3), suggesting that bulk RNA-based test shows over-fitting. By comparison, the correlations between RNA levels detected by scRNA-seq and RNA FISH, we observed that the gene-gene relationship had been dramatically changed if imputation is done by MAGIC or scImpute. The direction of a change is different: MAGIC tends to overestimate the correlation between genes, while scImpute tends to underestimate it, especially when the share of the dropouts is high. This observation is in line with the previously expressed concern regarding the circularity coming from solely internal information being used for imputation, which artificially amplifies the signal and leads to inflated correlations between genes.

Epi-Impute allows capturing even low-expressed regulatory genes facilitating downstream analysis such as clustering and inferring gene regulatory networks at the single-cell level. Clustering based on imputed TF expression shows very distinct clusters, which might look artificial since progenitor cells are expected to form a continuous trajectory in a real biological system. On the other hand, in the studied data set, the cells were experimentally pre-selected by FACS using surface markers, which might reduce the number of cells with a transient phenotype.

Overall, we recommend using Epi-Impute to improve recovery of dropouts, especially in the case when the input scRNA-seq matrix is highly sparse, or genes of potential interest for the researcher are expected to be low-expressed. Both types of data, scRNA-seq and scATAC-seq, should be available for the same biological sample either from a multiomix protocol or from separate experiments. In the latter case, preliminary annotation of cell types should be performed in both data sets. A set of potential enhancers will also be beneficial for the application of Epi-Impute, although it is not required. Since Epi-Impute does not show a tendency to generate a large number of false positives during imputation as other methods are prone to do [17], we recommend using it in the case when the specificity of the results is critical, for example, when looking for specific markers of a novel cell type or condition. Summing up, we believe that Epi-Impute is a helpful tool capable of recovering the expression landscape even with a high share of dropouts.

## 4. Materials and Methods

### 4.1. Datasets and Data Processing

We used nine datasets for methods benchmarking. A complete list of the scRNA-seq and scATAC-seq datasets is provided in Supplementary Table S1. If not explicitly stated otherwise, all quality metrics and rates were estimated on an aggregated dataset. For datasets aggregation, we used R version 4.0.0. (the details on aggregation are available on GitHub). We used gene annotations from the GENCODE releases vM20 (mm10), v30 (hg38) and galGal5 reference build. Row reads were aligned with bowtie2 (parameters: --very-sensitive and -X {2000). Only aligned reads with MAPQ>30 were kept for the downstream analysis. Following the recommendations in [19], all scATAC-seq reads aligned to the Watson strand were offset by *+4 bp* and all reads aligned to the Crick strand were offset *–5  bp*, since Tn5 transposase binds as a dimer and inserts two adaptors separated by *9 bp* [31]. Final scATAC-seq profiles are available at UCSC Genome Browser track hub (https://de.cyverse.org/dl/d/11108AB1-4B39-4A10-9864-B1C76E4EA64B/hub.txt, accessed on 15 March 2023).

### 4.2. Regulatory Regions

We used transcription start sites (TSS) from the FANTOM5 Human Promoter Expression Atlas [32], as well as enhancers and TSS-enhancers associations from the FANTOM5 Transcribed Human Enhancer Atlas [29]. For *Gallus gallus* and *Mus musculus* promoters regions were taken from the Eukaryotic Promoter Database (EPD) [33] and enhancer regions from EnhancerAtlas 2.0 [34].

### 4.3. Methods Benchmarking

The list of methods used for benchmarking is provided in Supplementary Table S2. We applied all methods to raw counts as suggested by the authors, except for DrImpute, for which, as per the documentation, we used $log_{2}\left(CPM+1\right)$ function, where CPM represents counts per million. Since, as usual, gene expression and chromatin accessibility were not obtained from the same cells, we performed a preliminary scRNA-seq and scATAC-seq integration using Seurat v4.0 R package [35] and thus were able to obtain cell annotations by transferring cell labels from the scRNA-seq to the scATAC-seq data set. For the benchmarking study, we used the following parameters suggested by authors for the best performance: SAVER *(all genes)*, MAGIC *(k = 12, t = 3)*, scImpute *(dropout threshold = 0.5 and the number of clusters equal to a number of FACS-defined cell types)*, DrImpute *(remaining zeros = 0)*, DCA (*hidden layer size = 32*). We run all methods with the default build-in parameters on 30 CPU (*Intel(R) Skylake Xeon(R) CPU E5-2620 v4 @ 2.10GHz*) or 1 GPU (*NVIDIA GeForce GTX 1080 Ti*) if no additional recommendations were provided. Execution times for each of the methods are presented in Supplementary Figure S1.

Since each of the data sets had unknown prior sparsity due to dropouts, only simulated dropouts were used to estimate the recovery performance of the methods. For this study, dropouts (25%, 50%, 75%, 95%) in scRNA-seq data were simulated by random sampling from a subset of all non-zero elements of the scRNA-seq matrix using Latin hypercube sampling in equal proportion for all cell types. Each time after the introduction of a share of dropouts to the dataset, joint co-embedding was performed using *Signac* R package [36] to obtain annotation for cells in ATAC-seq data.

We estimated *True positive (TPR)* and *False positive (FPR)* rates for all the methods using two alternative ways of defining *True positive* and *True negative* classes. The first method defines classes based on the expression of cell-type-specific positive and negative surface markers defined based on surface markers (“Marker test”, Supplementary Table S3). The alternative approach to defining *True positive* and *True negative* classes is to consider the most expressed genes in the bulk RNA-seq as a positive class and zero-expressed genes as a negative class for the matching cell population. For human hematopoiesis, we used a bulk RNA-seq dataset from [12] (“Bulk RNA-seq test”).

To further validate that Epi-Impute could capture the primary distribution of gene-gene relationship, we used RNA FISH [14] and Drop-seq data (8498 cells) in the melanoma cell line (WM989-A6) [28]. RNA FISH provides an estimate of the “ground truth” Person correlation between the fluorescence levels of the housekeeping genes in cell colonies (n = 17,095). scATAC-seq data for melanoma cell populations were obtained from SKCM cells [37].

### 4.4. Downstream Analysis

For the downstream analysis, data were normalized by SCTransform normalization from Seurat v.4.1.1 R package [35], R version 4.2.1. The most variable genes after imputation were used to generate a UMAP using Seurat v.4.1.1 R package. For visualization, min.cutoff was applied.

PAL analysis was carried out using the Oncobox web application [38]. For this, the geometric mean expression was obtained for each RNA in the target cluster and the comparison cluster.

## Figures and Tables

**Table 1:** Preservation of the “ground truth” gene-gene relationship (obtained by RNA FISH [14]) in the Drop-seq data  [28] after imputation. Person correlation of expression levels between housekeeping genes *BABAM1* and *LMNA* for the scRNA-seq data (n = 8498) with varying ratios of added dropouts (raw, 25%, 50%, 75% and 95%). The top 5 methods having the closest to the “ground truth” correlation, obtained using RNA FISH data (r=0.67) are marked in bold. * “Ground truth” correlation based on a RNA FISH data between *BABAM1*/*LMNA* (n = 17,095) is r=0.67  [14].

Method	Raw *	25%	50%	75%	95%
Epi-Impute	**0.68**	**0.67**	**0.67**	**0.65**	**0.61**
SCRABBLE	0.65	0.62	**0.64**	**0.61**	**0.52**
MAGIC	0.96	0.96	0.97	0.98	1.00
scImpute	0.33	0.27	0.22	0.23	0.26
Seurat v4	**0.67**	**0.68**	**0.66**	**0.72**	**0.73**
SAVER	**0.69**	**0.70**	**0.69**	**0.71**	**0.72**
DrImpute	**0.68**	0.71	0.77	**0.81**	0.83
coupledNMF	**0.66**	**0.69**	0.55	0.52	**0.59**
DCA	0.64	**0.65**	**0.74**	0.89	0.91

## Data Availability

Epi-Impute is available at https://github.com/raevskymichail/epi-impute (accessed on 15 March 2023) as an R package.

## Supplementary Materials

The supporting information can be downloaded at: https://www.mdpi.com/article/10.3390/ijms24076229/s1.
