# Supplementary material for: Epi-Impute: Single-Cell RNA-seq Imputation via Integration with Single-Cell ATAC-seq
Source: Int J Mol Sci. 2023 Mar 25;24(7):6229. doi: 10.3390/ijms24076229 (PMC10094055; doi:10.3390/ijms24076229)
Supplement: Supplementary file 1 [file ijms-24-06229-s001.zip › ijms-2116833-supplementary.pdf]

Supplementary Information

Table S1. Data sets analysed in the current study.

Data Type	Organism	Source	Link
scRNA-seq + scATAC-seq	*Gallus gallus*	Williams R. M. et al. (2019)	https://www.ncbi.nlm.nih.gov/geo/query/acc.cgi?acc=GSE131688
scRNA-seq	*Mus musculus*	Schaum N. et al. (2018)	https://tabula-muris.ds.czbiohub.org/
sci-ATAC-seq	*Mus musculus*	Cusanovich D. et al. (2018)	http://atlas.gs.washington.edu/mouse-atac/
scRNA-seq	*Homo sapiens*	Pellin D. et al. (2019)	https://www.ncbi.nlm.nih.gov/geo/query/acc.cgi?acc=GSE117498
scATAC-seq	*Homo sapiens*	Buenrostro J. D. et al. (2018)	https://www.ncbi.nlm.nih.gov/geo/query/acc.cgi?acc=GSE96772
scRNA-seq	*Homo sapiens*	10X Genomics Portal	https://support.10xgenomics.com/single-cell-gene-expression/datasets/2.1.0/pbmc8k
scRNA-seq	*Homo sapiens*	10X Genomics Portal	https://support.10xgenomics.com/single-cell-gene-expression/datasets/2.1.0/pbmc4k
scATAC-seq	*Homo sapiens*	10X Genomics Portal	https://support.10xgenomics.com/single-cell-atac/datasets/1.1.0/atac_v1_pbmc_10k
scRNA-seq + scATAC-seq	*Homo sapiens*	Yost K.E. et al. (2019)	https://www.ncbi.nlm.nih.gov/geo/query/acc.cgi?acc=GSE123814
scRNA-seq + scATAC-seq *	*Homo sapiens*	Jason D.Buenrostro et al. (2018)	https://doi.org/10.1016/j.cell.2018.03.074

* not used in an aggregated dataset.

Table S2. Imputation methods used in the current study.

Method	Source	Link
SCRABBLE	Tao Peng, et al. (2019)	https://genomebiology.biomedcentral.com/articles/10.1186/s13059-019-1681-8
MAGIC	David van Dijk, et al. (2018)	https://www.cell.com/cell/fulltext/S0092-8674(18)30724-4
scImpute	Wei Vivian Li, Jingyi Jessica Li. (2018)	https://www.nature.com/articles/s41467-018-03405-7
SAVER	Mo Huang, et al. (2018)	https://www.nature.com/articles/s41592-018-0033-z
DrImpute	Wuming Gong, et al.	https://bmcbioinformatics.biomedcentral.com/articles/10.1186/s12859-018-2226-y
DCA	Gökcen Eraslan, et al. (2019)	https://www.nature.com/articles/s41467-018-07931-2
coupledNMF	Zhana Duren, et al. (2018)	https://www.pnas.org/doi/full/10.1073/pnas.1805681115
Seurat V4	-	-

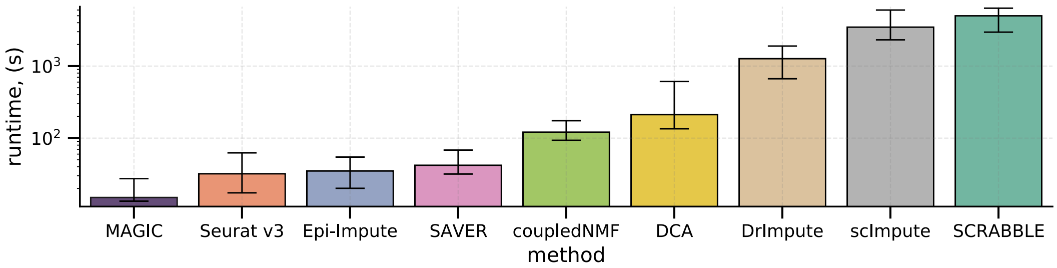

Supplementary Figure S1. Execution time spend on Pellin et al. (2019) dataset under the same conditions: 5 run per each method on 4 *Intel(R) Skylake Xeon(R) CPU E5-2620 v4 @ 2.10GHz* and 128GB *DIMM DDR4 Synchronous 2133 MHz*.

Table S3. List of cell-type-specific surface markers for the populations of analysed human hematopoetic progenitors [36].

Acronym	Cell-Type	Positive Markers	Negative Markers
HSC	hematopoietic stem cell	**CD34**(+)<br>**CD90/THY1**(+)<br>**CD59**(+)<br>**CD49f/ITGA6**(+)	**Lineage***(-)<br>**CD38**(-)<br>**CD45RA/PTPRC**(-)
CMP	common myeloid progenitor	**CD34**(+)<br>**CD38**(+)<br>**CD135/FLT3**(+)	**Lineage***(-)<br>**CD7**(-)<br>**CD10/MME**(-)<br>**CD45RA/PTPRC**(-)
GMP	granulocyte-monocyte progenitor	**CD34**(+)<br>**CD38**(+)<br>**CD45RA/PTPRC**(+)	**Lineage***(-)<br>**CD7**(-)<br>**CD10/MME**(-)<br>**CD135/FLT3**(-)

* Lineage markers are **CD15/FUT4**(+), **CD71/TFRC**(+), **CD41/ITGA2B**(+) and **CD19**(+).

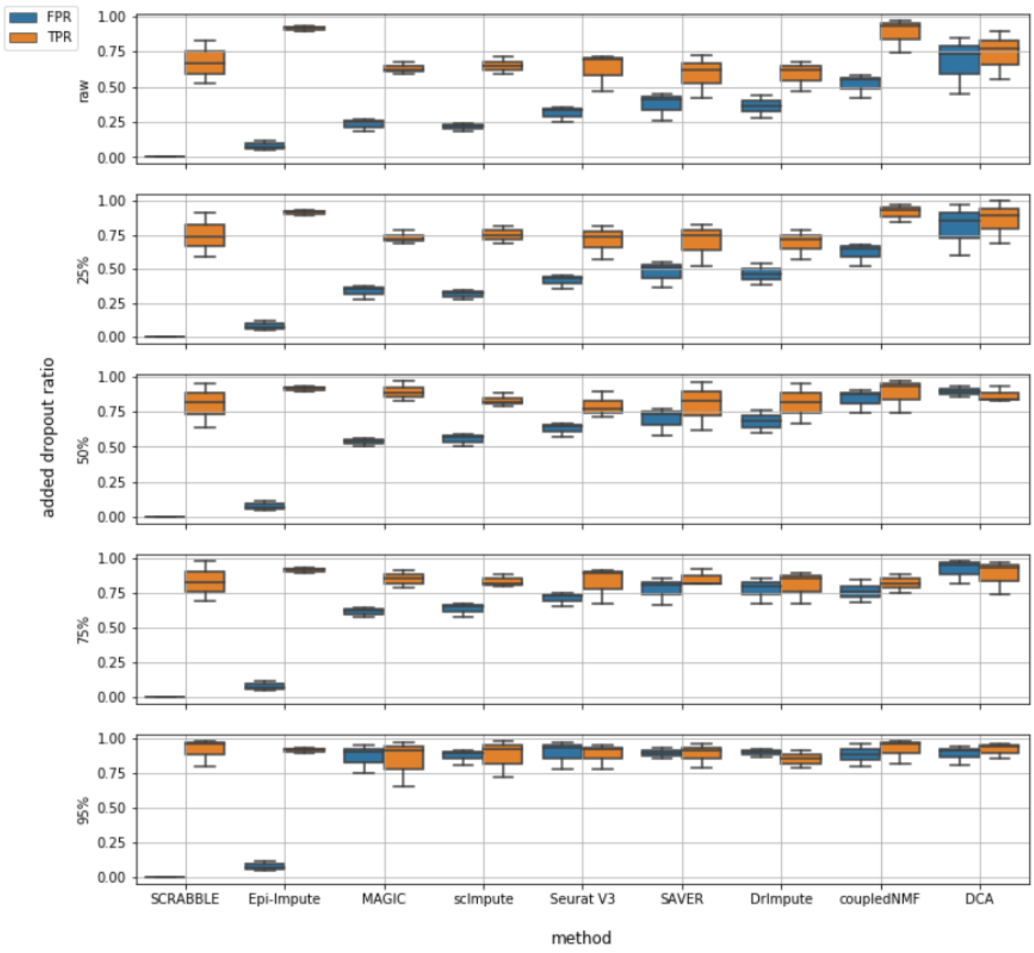

Supplementary Figure S2. True positive (TPR) and false positive rates (FPR) estimated based on bulk RNA-seq data. (**Top**) to (**bottom**): TPR and FPR for each of the method tested on the series of data sets with given ratio of simulated drop-outs. Each boxplot denotes a distribution of the performance metric obtained across all cell types presented in the series of data sets.

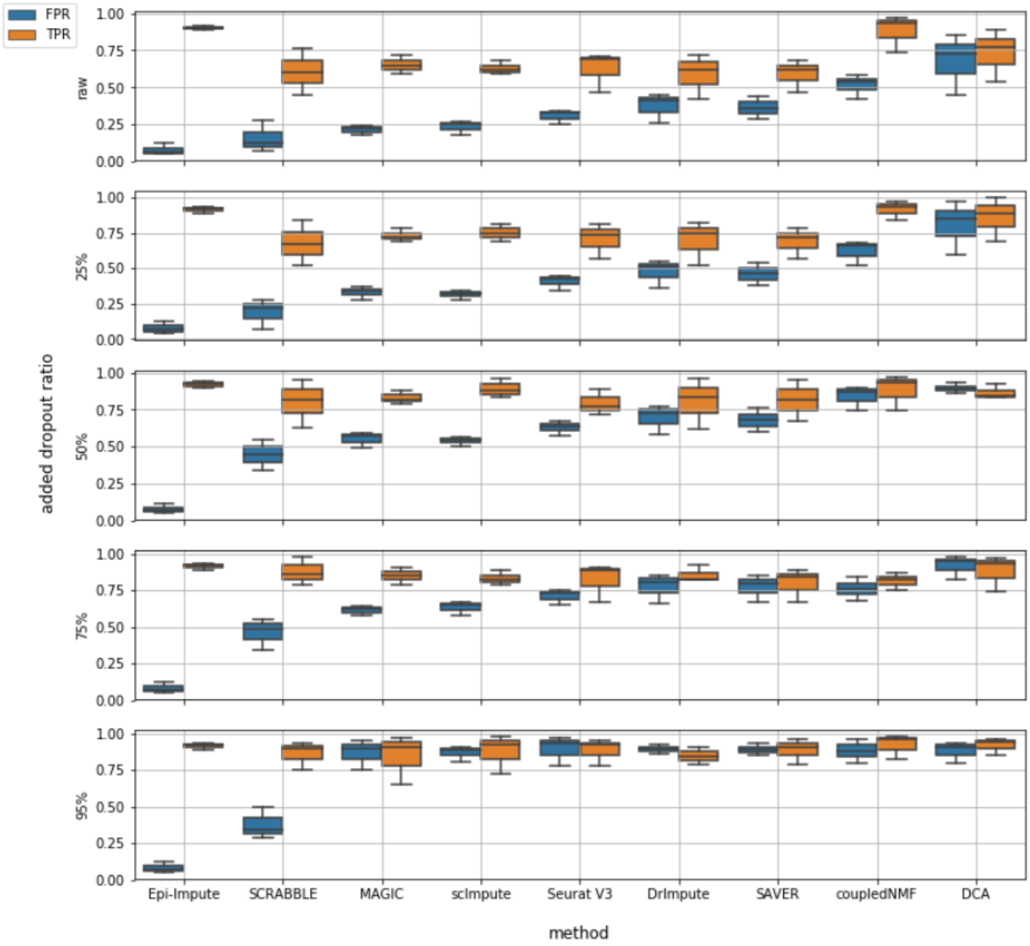

Supplementary Figure S3. True positive (TPR) and false positive rates (FPR) estimated based on the recovery of cell-type surface markers on an aggregated dataset, collected from Tabula Muris, PBMC 10x datasets and some GEO datasets. (**Top**) to (**bottom**): TPR and FPR for each of the method tested on the series of data sets with given ratio of simulated drop-outs. Each boxplot denotes a distribution of the performance metric obtained across all cell types presented in the series of data sets.

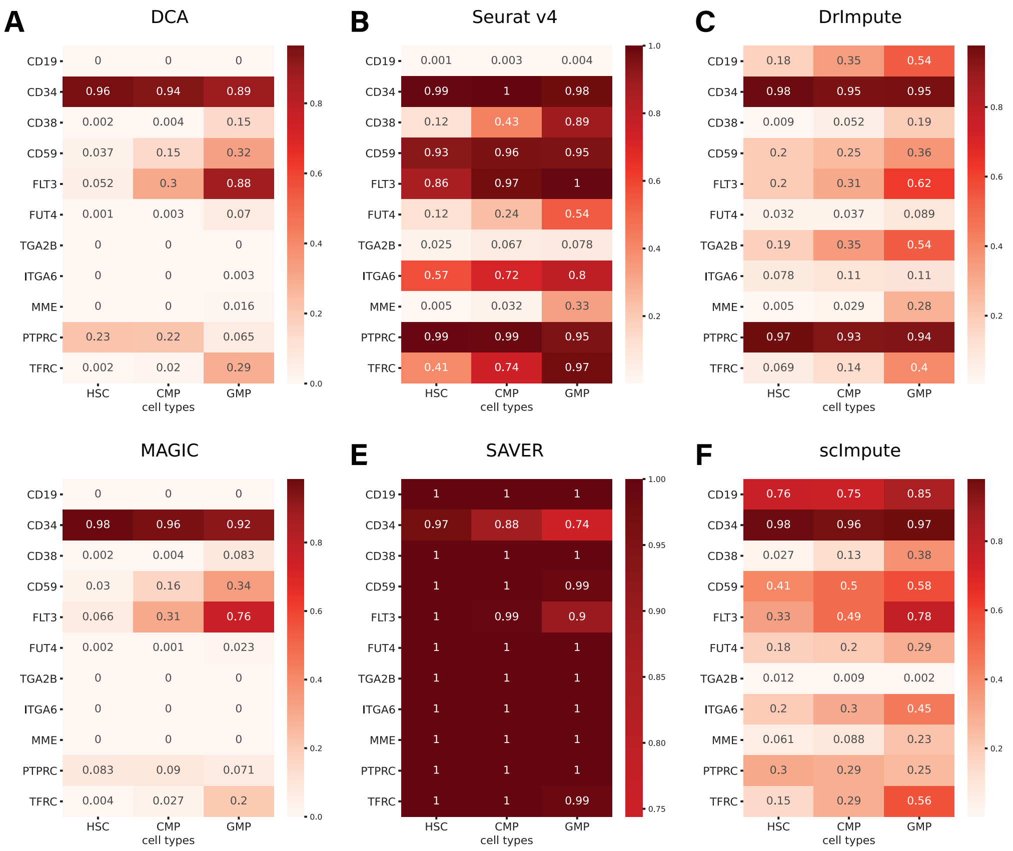

Supplementary Figure S4. A proportion of cells having an expression of known for hematopoiesis cell-type-specific surface markers after imputation of Pellin D. at el. (2019) dataset with various method: (**A**) DCA, (**B**) Seurat v4, (**C**) DrImpute, (**D**) MAGIC, (**E**) SAVER, (**F**) scImpute.

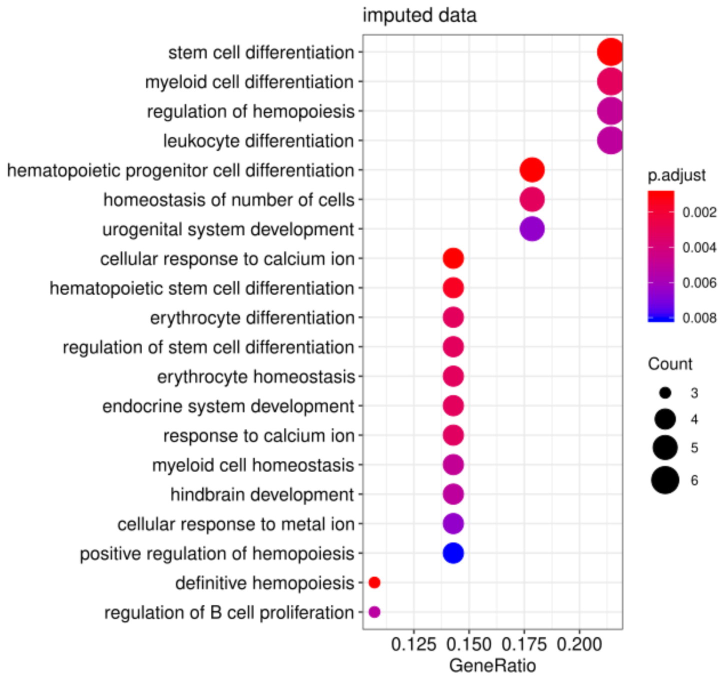

Supplementary Figure S5. GO Biological Process Enrichment for the most deferentially expressed transcription factors in common myeloid progenitors (CMP).

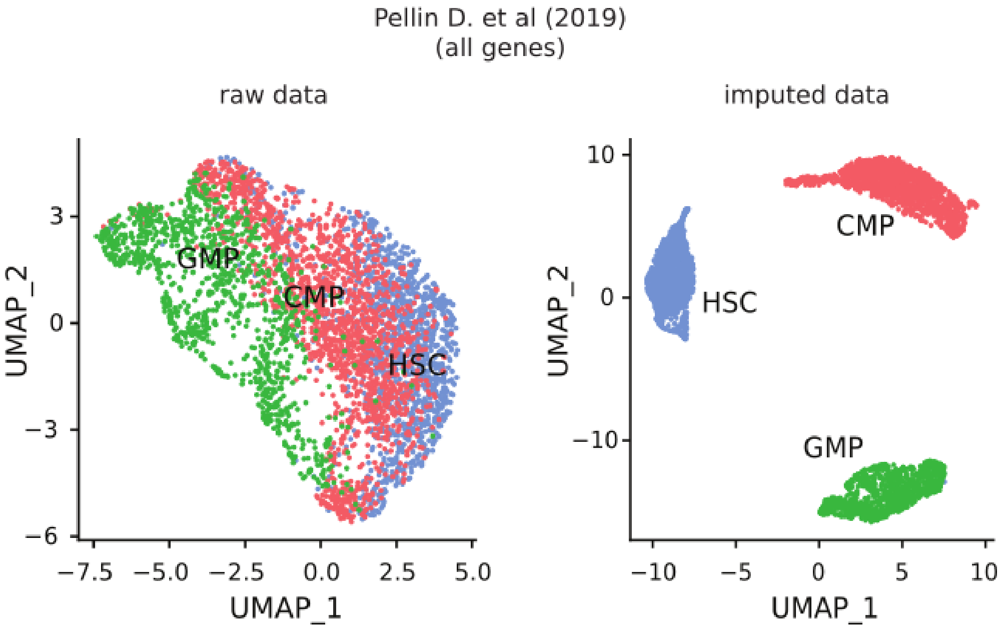

Supplementary Figure S6. UMAP clustering of all gene expression for raw data (**left**) and imputed with Epi-Impute (**right**).

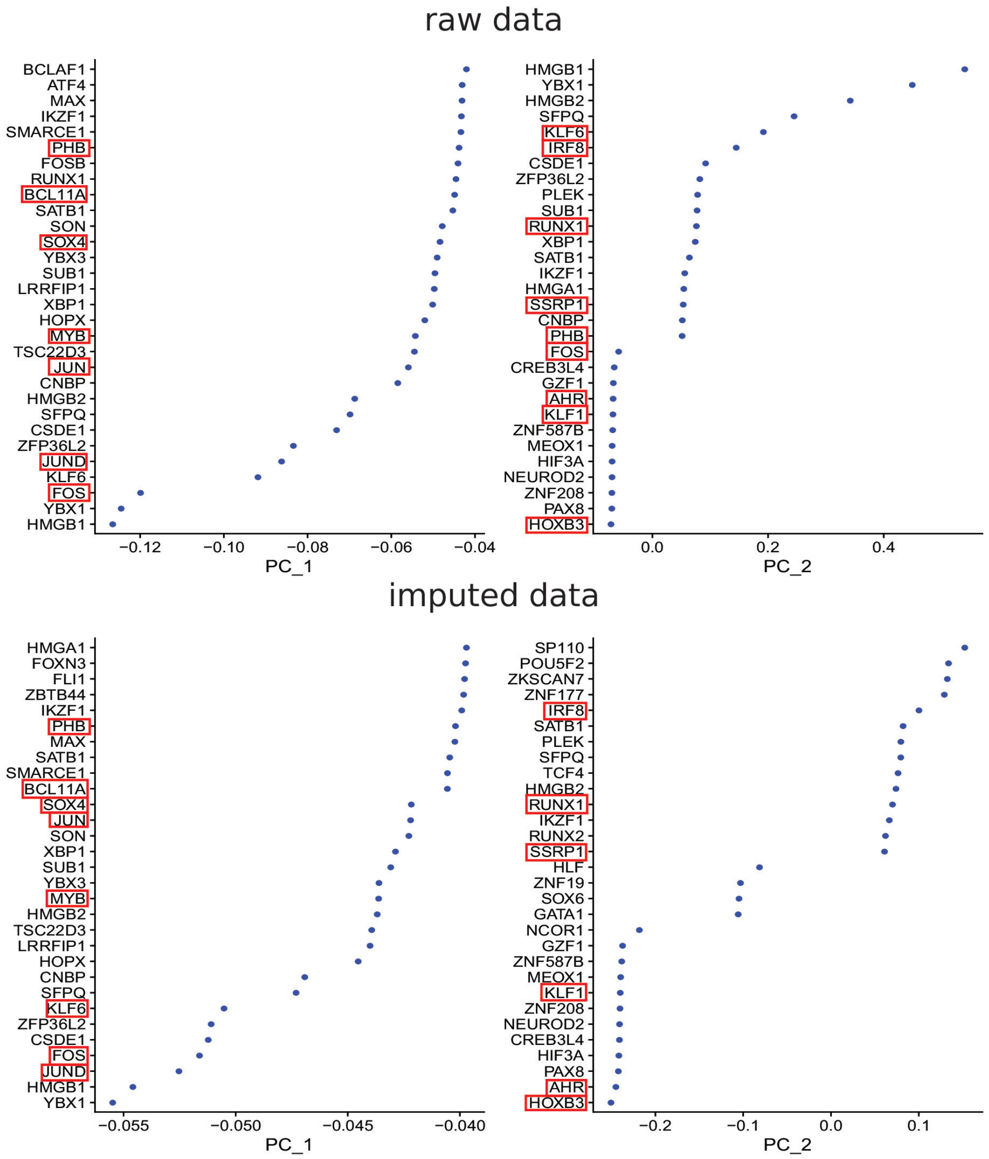

Supplementary Figure S7. PCA dimension loadings used for UMAP clustering of the transcription factors expression for raw data (**top**) and imputed with Epi-Impute (**bottom**). Genes which remained after imputation are marked with red.

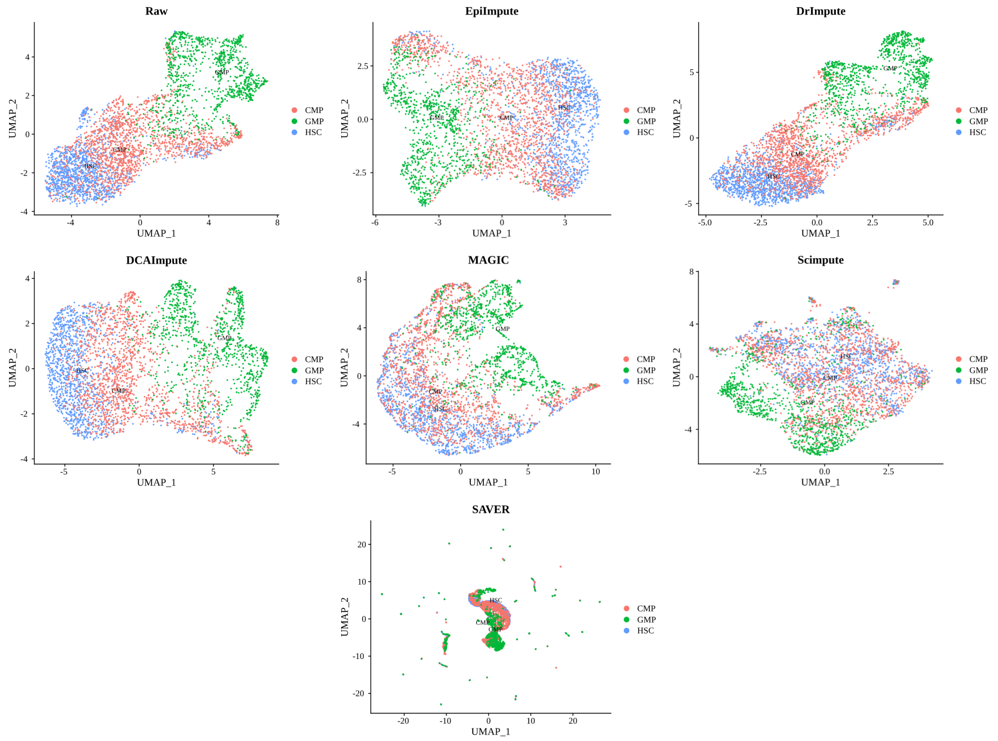

Supplementary Figure S8. UMAP clustering of six imputation methods and raw data for Jason D.Buenrostro et al. (2018) dataset.

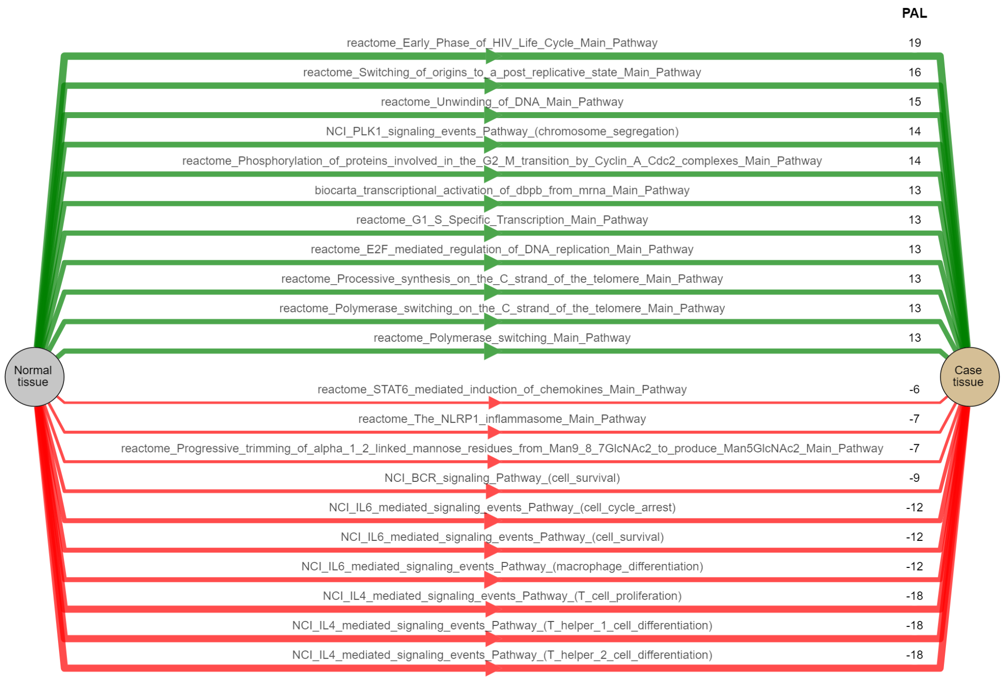

Supplementary Figure S9. PAL analysis of imputed cells via Epi-impute for a cluster 4 vs clusters 1+5 (Jason D.Buenrostro et al. (2018) dataset).

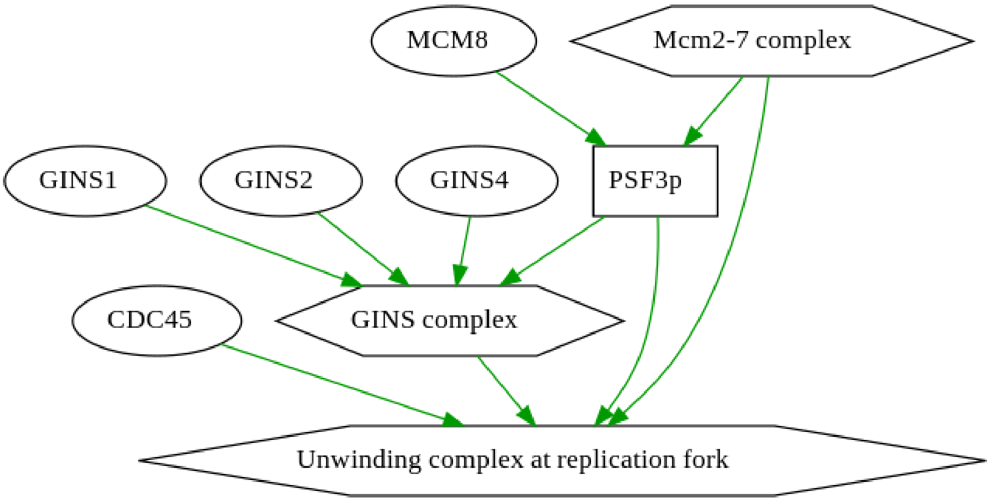

Supplementary Figure S10. Graphic scheme for "Reactome Unwinding of DNA_Main Pathway".

Table S4. Mean Silhouette Coefficient of six imputational methods and raw data for or Jason D.Buenrostro et al. (2018) dataset.

Data	Mean Silhouette Coefficient
Raw	0.0295
Epi-impute	0.0285
Magic	0.0187
DrImpute	0.0191
DCA	0.0589
Saver	0.0043
ScImpute	0.0151
